# MPO-ANCA associated vasculitis with mononeuritis multiplex following influenza vaccination

**DOI:** 10.1186/s13223-017-0222-9

**Published:** 2017-12-13

**Authors:** Stefanie Eindhoven, Jolien Levels, Margriet Huisman, Koos Ruizeveld de Winter, Virgil Dalm, Rehmat Alwani

**Affiliations:** 1 Department of Internal Medicine, IJsselland Hospital, Capelle aan den IJssel, The Netherlands; 2 Division of Rheumatology, Department of Internal Medicine, IJsselland Hospital, Capelle aan den IJssel, The Netherlands; 3 Department of Pathology, Pathan, Rotterdam, The Netherlands; 4 Division of Clinical Immunology, Department of Internal Medicine, Erasmus Medical Centre, Rotterdam, The Netherlands; 5 Department of Immunology, Erasmus Medical Centre, Rotterdam, The Netherlands; 6 Department of Internal Medicine, Room F104, IJsselland Hospital, P.O. Box 690, 2900 AR Capelle aan den IJssel, The Netherlands

**Keywords:** Influenza vaccination, Anti-neutrophil cytoplasmic antibody (ANCA), Vasculitis

## Abstract

**Background:**

Although influenza vaccines are generally safe and effective, a variety of autoimmune phenomena have been reported after vaccination over the past years, such as Guillain–Barre syndrome, rheumatoid arthritis, pemphigus vulgaris, psoriasis, giant cell arteritis and anti-neutrophil cytoplasmic antibody (ANCA) associated vasculitis (AAV).

**Case report:**

We describe the case of a 67-year old man who presented with a myeloperoxidase-ANCA associated vasculitis with renal involvement and mononeuritis multiplex after seasonal influenza vaccination. He was initially treated with intravenous cyclophosphamide and high-dose prednisolone followed by maintenance treatment consisting of azathioprine and prednisolone.

**Conclusion:**

We hypothesize that seasonal influenza vaccination triggered a systemic immune response in a susceptible patient to develop AAV with renal involvement and vasculitic neuropathy. In general, seasonal influenza vaccinations are considered to be safe, however, clinicians should be aware of this rare phenomenon.

## Background

Seasonal influenza vaccination is widely used in the western world, particularly in the elderly population, chronically ill patients, immunocompromised subjects and health care workers to provide protection against influenza and its potential secondary complications. Although influenza vaccines are generally safe and effective, a variety of autoimmune phenomena have been reported after vaccination over the past years, such as Guillain–Barre syndrome, rheumatoid arthritis, pemphigus vulgaris, psoriasis, Henoch–Schönlein purpura, polymyalgia rheumatic, giant cell arteritis and anti-neutrophil cytoplasmic antibody (ANCA) associated vasculitis (AAV) [1–5]. AAV is a rare disease with an overall annual incidence of 13–20 cases per million individuals, with a peak incidence of only 65 per million/year in those aged 65–74 years [6, 7]. AAV is a small to medium vessel vasculitis, and comprises three syndromes: granulomatosis with polyangiitis (GPA), microscopic polyangiitis (MPA) and eosinophilic granulomatosis with polyangiitis (EGPA) [8]. Here we describe a case of a patient presenting with AAV after influenza seasonal vaccination.

## Case report

A 69-year-old Caucasian male patient, with no significant medical history, was evaluated in the IJsselland hospital. Since 8 weeks he had been suffering from fatigue, intermittent fever and night sweats, weight loss (4 kg) and macroscopic haematuria. In addition, he complained of progressive muscle weakness in hands and legs as well as stiffness, numbness and tingling of both hands. He had gradually lost the ability to walk due to a lack of strength in his legs. Clinical symptoms started 2 weeks after influenza vaccination. Over the past 3 years he received influenza vaccinations twice without any complications.

Physical examination revealed weakness in both his upper legs and arms and a reduced sense of feeling in his right arm. Also, both hands showed signs of muscular atrophy of the thumb muscles with flattening of the ball. The tendon reflexes at his legs were normal.

**Fig. 1:**
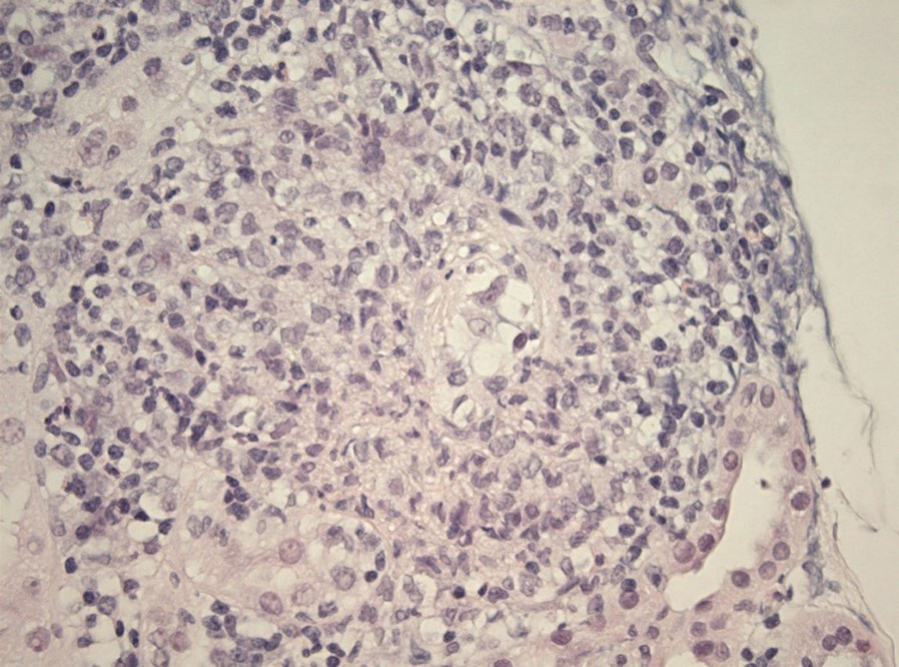
Light microscopy imaging of the renal biopsy demonstrating a small vessel vasculitis. Original magnification ×400, using Haematoxylin–eosin stain

**Fig. 2:**
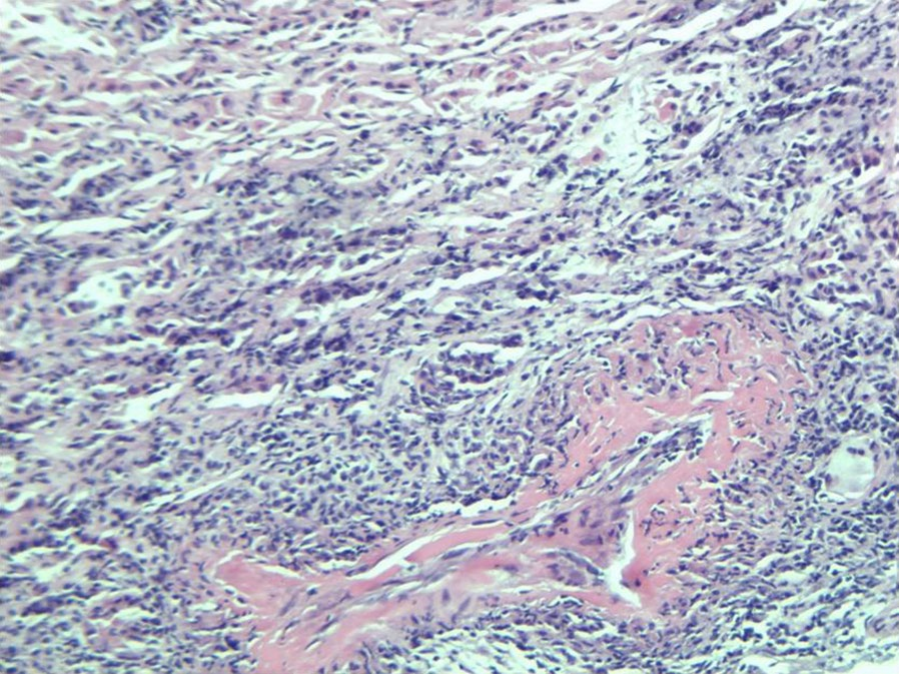
Light microscopy imaging of the renal biopsy demonstrating a medium sized vasculitis. Original magnification ×100, using Haematoxylin–eosin stain

Blood tests showed raised levels of erythrocyte sedimentation rate (ESR) and C-reactive protein (CRP), a mild normocytic anaemia, leukocytosis and normal renal function (creatinine 87 μmol/l) (Table 1). At admission the urinalysis demonstrated no active urinary sediment. Three weeks after hospital admission, serology revealed a positive perinuclear anti-neutrophil cytoplasmic antibody (pANCA) with positive myeloperoxidase (MPO)-ANCA at a titer of 46 E/ml (reference < 3.5 E/ml). It was unknown if the patient was MPO-ANCA positive in the past. Imaging studies with computed tomography scan (CT scan) of chest and abdomen, magnetic resonance imaging (MRI) of head and neck, and positron emission tomography with 2-deoxy-2[fluorine-18]fluoro-d-glucose with computed tomography (18F-FDG PET/CT) revealed no abnormalities. No increase in cell count was seen during lumbar puncture. Cultures of cerebrospinal fluid, blood and urine as well as a tuberculin skin test and a quantiferon test were negative. Electromyography (EMG) showed decreased compound muscle action potentials (cmap) of the peroneal nerves of both legs and the tibial nerve of the right leg. All the additional research was done before administration of prednisolone. During hospitalization kidney function deteriorated, with a maximum serum creatinine level of 178 µmol/l (upper limit of reference value 104 μmol/l). Three weeks after hospital admission, the urinalysis became deviant with red blood cells (2+) and protein (2+), dysmorphic erythrocyes and/or red cell casts were not observed. Renal biopsy was conducted which revealed a small and medium-size vasculitis and tubulo-interstitial infiltration (Figs. 1 and 2). Three out of ten glomeruli showed glomerulosclerosis. Additional immunofluorescence with IgA, IgG, IgM, C3 and C1q were negative.

**Table 1:** Laboratory data of the patient on admission

		Reference value
Hematology		
Hemoglobin (mmol/l)	7.6	8.5–11.0
Mean corpuscular volume (fl)	81	80–100
Hematocrit (l/l)	0.37	0.40–0.51
Red blood cells	4.7 × 10E12/l	4.6–6.1 × 10E12/l
White blood cells	20.6 × 10E9/l	4.2–9.1 × 10E9/l
Neutrophils	17.0 × 10E9/l	1.5–9.0 × 10E9/l
Lymphocytes (%)	7	20–50
Monocytes (%)	9	2–10
Eosinophils (%)	1	< 5
Basophils (%)	0	< 2
Platelets	397 × 10E9/l	165 × 10E9/l
Biochemistry/serology		
Creatinine (μmol/l)	87	59–104
Sodium (mmol/l)	135	135–145
Potassium (mmol/l)	3.8	3.5–5.0
Blood urea nitrogen (mmol/l)	6.0	2.5–7.5
C-reactive protein (mg/l)	163	< 5.0
Erythrocyte sedimentation rate (mm/h)	103	< 15
Albumin (g/l)	26	35–52
Ferritin (μg/l)	710	25–250
Vitamin B12 (pmol/l)	380	140–500
Folic acid (nmol/l)	10.3	10.4–42.4
Total bilirubin (μmol/l)	27	< 21
Gamma glutamyl transferase (E/l)	240	< 55
Alkaline phosphatase (E/l)	230	< 115
Aspartate aminotransaminase (E/l)	28	< 35
Alanine aminotransaminase (E/l)	34	< 45
Lactate dehydrogenasis (E/l)	183	< 248
Creatine kinase (E/l)	38	< 171
MPO-ANCA (E/ml)	46	< 3.5
PR3-ANCA (E/ml)	0.3	< 7.0

Urinary sediment		
Protein	2+	
Hemoglobine	2+	
Red blood cells	4–10/HPF	
Dysmorphic red blood cells (%)	< 40

Our patient was diagnosed having MPO-ANCA associated vasculitis with involvement of kidneys and peripheral nerves one month after admission to the hospital. Initial symptoms started two weeks after seasonal influenza vaccination. Because of rapidly progressive disease therapy was initiated with high dosages of intravenously administered prednisolone (1000 mg) for three consecutive days followed by oral prednisolone in tapering regimen, in combination with intravenous pulse cyclophosphamide (700 mg: 10 mg/kg adjusted for age and renal function) and trimethoprim/sulfamethoxazole (480 mg daily) for *Pneumocystis jiroveci* pneumonia prophylaxis, according to the consensus regimen of the European vasculitis study group [9]. The cyclophosphamide treatment was continued for three months, resulting in remission of the disease process. Levels of ESR and CRP dropped and renal function stabilized (serum creatinine levels 140–150 µmol/l). He was discharged from the hospital to a rehabilitation centre for further recovery. Patient continued on azathioprine for another 18 months. Remaining symptoms one year after initiation of treatment consist of distal sensory loss of hands and feet and loss of fine motor skills of the hands.

## Discussion

In this report, we described a patient with MPO-ANCA vasculitis with renal involvement and mononeuritis multiplex two weeks after seasonal influenza vaccination. Since the patient showed overlapping sets of clinical features it was difficult to diagnose the exact subtype of AAV [10, 11]. Currently, a handful of AAV cases have been reported in temporal association with influenza vaccination [12–17]. Recently, The Brighton Collaboration Vasculitis Working Group published a systematic literature review on adverse events following immunization (AEFI) suggesting an association between influenza vaccination and vasculitis, however no evidence is found to confirm this association yet [18]. The temporal relationship with the influenza vaccine could have been completely coincidental in our case. Several other etiological factors, such as exposure to silica, viral or bacterial infections, medication and genetic susceptibility have also been correlated to AAV [19, 20].

Vasculitic neuropathy is characterized by a necrotizing vasculitis involving the small arterioles of peripheral nerves. Mononeuritis multiplex is a painful, asymmetrical, asynchronous sensory and motor peripheral neuropathy involving isolated damage to at least two separate nerve areas. It can be distributed bilaterally, distally and proximally throughout the body, as we describe in our patient [21]. Hadden et al. reported several case reports with vasculitic neuropathy after influenza vaccination [22]. Mononeuritis multiplex was not proven histopathologically in our patient. However, the progressive neurological impairment was highly suggestive of mononeuritis multiplex, especially in combination with the results of the performed EMG as well as the histopathological proven renal vasculitis (level 2 evidence according to The Brighton Collaboration Vasculitic Peripheral Neuropathy Working Group) [22].

The exact etiology of post-influenza vaccination vasculitis is unknown. Several possible underlying mechanisms have been postulated, such as molecular mimicry, autoimmune syndrome induced by adjuvants (ASIA) and viral ribonucleic acid (RNA). In molecular mimicry, a microbial/foreign antigen shares structural similarities with self-antigens. Prolonged inflammatory responses to these foreign antigens can therefore induce autoimmunity syndromes in predisposed individuals [23–25]. A second possible mechanism is ASIA. Adding an adjuvant to a vaccine antigen leads to several advantages, including dose sparing and the introduction of a more rapid, broader and strong immune response. However, disadvantages of these adjuvants have been described as well. The pathogenesis of the ASIA syndrome is founded on the hypothesis that an exposure to an adjuvant may trigger the development of an autoimmune disease [26, 27]. The influenza vaccination our patient received was the third vaccination in three years. The first two years he had no complications after the vaccination. In 2011, his first influenza vaccine contained 4 viral types: A/California/7/09 A (H1N1), A/Perth/16/2009 (H3N2), A/Victoria/2010/2009 and B/Brisbane/60/08. In 2012, his second influenza vaccine contained the same California type, but also contained B/Victoria/361/2011 (H3N2) and B/Wisconsin/1/2010. His third vaccination consisted of the same California and Victoria types and of B/Massachussets/02/2012. Unfortunately, we were not able to obtain information on whether these vaccines were adjuvanted or not. According to the ASIA criteria defined by Alijotas-Reig, our patient met three major criteria, including exposure to an external stimulus, minimum latency time of days and muscle weakness [28]. Recently, another possible mechanism has been proposed regarding influenza vaccines containing viral RNA which may increase the production of Proteinase 3 (PR3-ANCA) and thus further contribute to the development of AAV following influenza vaccination [17].

Several clinical trials have investigated the relapse rate of AAV after influenza vaccination. Overall, influenza vaccination does not seem to increase the relapse rate in patients with pre-existent AAV in remission [29, 30]. Furthermore, the disease free survival was lower in patients not vaccinated [30]. A recent report showed no changes in auto-antibody levels after influenza vaccination in a small group of healthy individuals [29]. However, one study found silent auto-antibody formation after influenza vaccination in healthy individuals, but this did not reach statistical significance and none of the participants had clinical symptoms of systemic autoimmune disease [31].

In this report we described another case suggesting a temporal relationship between influenza vaccination and AAV with mononeuritis multiplex. However, existing literature does not support a causative link between vaccination and vasculitis yet. Further research is needed to evaluate this potential causal relationship, especially given the extent of influenza vaccinations administered to the peak age incidence population on an annual basis. In general, seasonal influenza vaccinations are considered to be safe, however, clinicians should be aware of this rare phenomenon.

## Abbreviations

pANCA: perinuclear anti-neutrophil cytoplasmic antibody
ANCA: anti-neutrophil cytoplasmic antibody
AAV: ANCA-associated vasculitis
GPA: granulomatosis with polyangiitis
MPA: microscopic polyangiitis
EGPA: eosinophilic granulomatosis with polyangiitis
EMG: electromyography
cmap: compound muscle action potentials
ESR: erythrocyte sedimentation rate
CRP: C-reactive protein
MPO-ANCA: myeloperoxidase anti-neutrophil cytoplasmic antibody
PR3-ANCA: proteinase 3 anti-neutrophil cytoplasmic antibody
CT scan: computed tomography scan
MRI: magnetic resonance imaging
18F-FDG PET/CT: positron emission tomography with 2-deoxy-2[fluorine-18]fluoro-d-glucose
ASIA: autoimmune syndrome induced by adjuvants
RNA: viral ribonucleic acid
